# Hospital Meetings

**Published:** 1904-04-30

**Authors:** 


### HOSPITAL MEETINGS.

### THE EVELINA HOSPITAL FOR SICK CHILDREN.

The annual meeting of the Governors of this hospital was held on Tuesday, April 26. Mr. Charles Wightman, chairman of the committee of management, presided, in the absence through illness of the Earl of Ancaster, the President.

The Chairman, in moving the adoption of the report, said that he might claim that the report and balance sheet for 1903 were very satisfactory. In spite of the large sums of money they had been compelled to spend on improvements during the year, they were enabled to close it with only a comparatively small deficit. Still they must remember that owing to the general depression it was far from likely that they would be able to raise so much money during the ensuing year, and he therefore urged upon them to do all they could for the hospital and to endeavour to enlist the aid and sympathy of friends in the neighbourhood and elsewhere. He could affirm with confidence that they were doing a good work in a very poor district. The Chairman then referred in feeling terms to the loss the hospital had sustained through the death of Mr. Charles Cotes.

The report was then seconded and adopted; and the usual votes of thanks having been passed, the proceedings terminated.

The report for 1903 showed that the year had ended with a deficit of £398. An increase in the receipts from legacies and donations had been received, including £250 from King Edward's Hospital Fund, £216 from the Sunday Fund, and £98 from the Saturday Fund. Six hundred and eighty-three in-patients were admitted during the year, the average time each child remained being 21 days; 4,738 new out-patients attended the hospital, their total attendances amounting to 24,757.

